# The transcriptional and cellular landscape of cognitive resilience to Alzheimer’s disease

**DOI:** 10.3389/fnmol.2025.1665802

**Published:** 2025-11-10

**Authors:** Nina Khera, Ravikiran M. Raju, Stuart A. Lipton

**Affiliations:** 1 Harvard College, Cambridge, MA, United States; 2 Picower Institute of Learning and Memory, Massachusetts Institute of Technology, Cambridge, MA, United States; 3 Division of Newborn Medicine, Boston Children’s Hospital, Harvard Medical School, Boston, MA, United States; 4 Biology of Adversity Project, Broad Institute of MIT and Harvard, Cambridge, MA, United States; 5 Department of Molecular & Cellular Biology, Neurodegeneration New Medicines Center, The Scripps Research Institute, La Jolla, CA, United States; 6 Department of Neurosciences, School of Medicine, University of California San Diego, La Jolla, CA, United States

**Keywords:** cognitive resilience, Alzheimer’s disease, transcription factor, MEF2C, Nrf2, REST (RE-1 silencing transcription factor)

## Abstract

It is estimated that 5%–40% of patients with pathological features of Alzheimer’s disease (AD) maintain normal cognitive health throughout their lifetimes, a phenomenon known as cognitive resilience. Studies have identified many factors that contribute to a patient’s capacity for resilience, with those that modulate gene expression being the most dynamic, adaptable, and potentially addressable as targets for future drug development. In patients cognitively resilient to AD and AD-related dementias (ADRD), transcriptional changes within specific cell types serve to preserve the processes most critical to cognitive function within each cell, exerting protective effects on other cell types as well via non-cell autonomous effects. Key themes in preserved cognitive function include maintenance of synaptic stability and function, dampening neuronal hyperexcitability, reducing misfolded protein accumulation, increasing myelination, and countering neuroinflammation. With future research on the most upstream and impactful transcriptional drivers, there lies immense potential for both therapeutics to address AD and a greater fundamental understanding of AD and the brain.

## 1 Introduction

Alzheimer’s Disease (AD), a disorder that causes synaptic dysfunction and neuronal cell death in the brain, is the primary cause of dementia in the elderly, a condition that impacts memory skills, language processing, and reasoning abilities (Alzheimer’s Association, 2024; Knopman et al., 2021). As the disease progresses, there are several neuropathological changes in the brain. Pathological hallmarks include accumulation of extracellular β-amyloid (Aβ) aggregates or plaques, and soluble oligomers of Aβ which are thought to disrupt synaptic transmission, damage neurons, and trigger inflammation (Wu T. et al., 2022). In patients without AD, normal tau protein plays a role in microtubule-mediated axonal transport in neurons; however, in AD, hyperphosphorylated tau misfolds to form intraneuronal tangles that disrupt both axonal transport and synaptic function (Medeiros et al., 2011; Ashraf et al., 2014; Ittner et al., 2010). In addition, many other less-prevalent proteins build up and/or lose function, including TDP-43 and α-synuclein protein, although the former is classically associated with frontotemporal dementia and amyotrophic lateral sclerosis, and the latter with Parkinson’s disease and Lewy body dementia (Meneses et al., 2021; Twohig and Nielsen, 2019). These changes have long been considered hallmarks of AD, and are often viewed as central drivers of neuronal cell death, and neurodegeneration. However, most authorities now agree that the principal correlate to cognitive decline is the loss of synapses in the brain and not the amount of these protein aggregates (Terry et al., 1991; DeKosky and Scheff, 1990; Mecca et al., 2022).

Intriguingly, a small number of adults seem to harbor these elements of AD-associated pathology, including substantial Aβ plaque and tau tangle accumulation, and yet remain cognitively intact and maintain normal or near-normal synaptic contacts (Montine et al., 2022). This phenomenon, dubbed “cognitive resilience,” challenges traditional models that view protein aggregation as necessary for AD and offers hope that despite pathological changes, patients can evade cognitive impairment (de Vries et al., 2024a; Montine et al., 2022).

The existence of these resilient individuals carries important implications for diagnosis and treatment. It adds complexity to current AD diagnosis methods involving measurement of pathology, and urges researchers to focus on more functional clinical endpoints for AD, such as scores on screening tests of cognitive performance like the Mini-Mental State Examination (MMSE) and the Montreal Cognitive Assessment (MoCA) or biomarkers of synaptic loss, such as positron emission tomography (PET) scans for the presynaptic marker SV2A, which has been reported to reflect synapse number (Arevalo-Rodriguez et al., 2021; Davis et al., 2021; Finnema et al., 2016). To date, this approach has not been widely promulgated, with the majority of AD research and clinical trials being focused on treatment to mitigate pathological burden of aggregated proteins.

Along these lines, a subfield of AD research is emerging to focus on understanding the origins and causes of resilience; this approach is accumulating evidence of genetic variants, lifestyle factors, and transcriptional changes that confer resilience to AD (Carrigan et al., 2023; de Vries et al., 2024a). Broadly, resilience-conferring factors appear to preserve brain reserve and neural plasticity with age. Current approaches to study resilience are multifold. For example, researchers have utilized mouse models of AD to identify molecular regulators of cognitive resilience. Primarily, this has been done by modulating the expression of putative resilience-conferring factors and assessing how these factors impact learning and memory in the face of AD pathology (Barker et al., 2021; Dunn et al., 2025; Roberts et al., 2024; Soni et al., 2024).

In the human context, resilience is often studied using analysis of neural tissue samples with imaging and structural quantification, as well as multiomics data collected from patients with and without resilience to AD. In these studies, resilience is often defined as greater than expected cognition or alternatively a slower than expected cognitive decline given the pathological state of the patient (Wagner et al., 2023; Mathys et al., 2023; Masurkar et al., 2024). Several of these studies analyze datasets such as the Religious Orders Study and Memory Aging Project (ROSMAP), the Mount Sinai Brain Bank, and the Nun Study (Bennett et al., 2018; Wang et al., 2018; Snowdon, 1997). PREVENT-AD, a dataset that collects multi omics and imaging data from those with a familial history of AD, is similar in nature, but is not a post-mortem dataset (Tremblay-Mercier et al., 2021). Moreover, there are a small number of other studies that test the efficacy of several lifestyle interventions in slowing the cognitive decline of live patients, such as the Finnish Geriatric Intervention Study to Prevent Cognitive Impairment and Disability (FINGER) Study performed by the Fingers Brain Health Institute in Finland (Kivipelto et al., 2013).

Research performed on data derived from twin studies in particular has shown that it is common for monozygotic twins to exhibit divergent cognition with identical pathology, emphasizing the role of non-genetic factors in predicting resilience (Gatz et al., 2006). However, genetics remain important, with other studies showcasing similar cognitive outcomes in monozygotic twins, finding that the proportion of explained variance in Alzheimer’s outcomes due to heritability was 74%, with the remaining 26% being attributed to the environment (Gatz et al., 1997). With regards to lifestyle, resilience is often cited as being associated with greater education levels, higher physical activity, changed diet, and other lifestyle factors that have been associated with improved aging and risk for other chronic conditions (Devanand et al., 2023; Wagner et al., 2023; Matyas et al., 2019; Masurkar et al., 2024).

Both genetic and lifestyle factors contribute significantly to resilience, and recent evidence suggests that both may converge on shared molecular mechanisms. In particular, a key mechanism that mediates resilience is the regulation of gene expression by transcription factors (TFs). Broadly, TFs encompass a family of proteins that respond to a number of different stimuli, and directly regulate gene expression by binding to DNA and coordinating as well as providing a buffer against alterations in gene transcription, thus capable of broadly altering cellular function across systems (Parisutham et al., 2025). Transcriptional regulation provides a highly dynamic layer of control, allowing the brain to respond to stressors in an adaptive manner. Transcriptional programs can shift over time and vary across cell types, allowing for a targeted system to preserve function in the face of aging and neurodegenerative pressure.

In this review, we explore how transcriptional regulators contribute to cell-type specific adaptations that preserve essential functions and network-level coordination in cognitive resilience. It is essential to first understand how a TF functions within the cell where it is expressed, as TFs first and foremost coordinate cell-specific actions that then interact within the complex cellular landscape of the brain. For this reason, we start by first giving an overview of each cell type and the TFs active within that a given cell type that contribute to resilience phenotypes. We then synthesize the findings from each cell type into common themes and principles the lead to a more unified understanding of master regulators and processes that are critical for inducing cognitive resilience. To identify candidate transcriptional regulators of cognitive resilience, we selected evidence through comprehensive searches of keywords related to resilience and transcriptional regulation, and included TFs in our review that were cited as modified in resilience. This heuristic synthesis of the literature provides a framework for understanding how transcriptional programs can contribute to cognitive resilience in individuals despite AD/ADRD neuropathology.

## 2 Transcriptional adaptations within cell types

Just as different cell types have varying functions within the brain, their functional loss and subsequent contribution to pathology during symptomatic AD varies greatly. These adaptations are likely the result of decades-long processes where cells attempt to compensate for building pathological stressors, and attempt to preserve their identity and function. As described below, several TFs emerge as pleiotropic regulators that enhance universal pathways conferring overall cell function and survival in the face of neuropathology. Other TFs specific to given cell types also emerge, preserving cell-type specific functions such as myelination capabilities in oligodendrocytes, anti-inflammatory and antioxidant properties in microglia, homeostatic mechanisms in astrocytes, and waste clearance in neurons. To better understand how cognitive resilience is facilitated by molecular regulators, we break down the next section by cell type, highlighting the transcriptional regulators and gene networks that preserve key functions within each.

### 2.1 Neurons

Neurons are the fundamental unit of the nervous system, receiving sensory information from the outside world, and communicating amongst themselves (termed association areas) and to motor outputs to influence behavior, and in this context cognitive function. In cognitive resilience, neurons mitigate degeneration through transcriptional changes that reduce protein aggregation, preserve synaptic stability and function, and reduce oxidative/nitrosative stress.

#### 2.1.1 Resilience mechanisms to avoid misfolded protein aggregation

In AD patients, there is significant Aβ deposition and tau tangle accumulation, among other proteins, disrupting neuronal communication and increasing degeneration (Ashraf et al., 2014). Cognitively resilient patients show upregulation of transcriptional networks that mitigate misfolded protein aggregation, synapse loss, and neuronal apoptosis or other forms of cell death. First, the TF REST plays a large role in conferring resilience to AD. Mechanistically, it targets genes in pathogenic pathways, downregulating pro-apoptotic genes such as death domain-associated protein (DAXX), forkhead box O3 (FOXO3), growth arrest and DNA damage inducible alpha (GADD45A) and caspase-9 (CASP9), genes whose protein products contribute to Aβ accumulation. Additionally, REST inhibits tau kinases, cyclin-dependent kinase (CDK5) and glycogen synthase kinase 3β (GSK3B) (Aron et al., 2023). REST achieves this repression by recruiting histone deacetylases to remove acetyl groups from chromatin, leading to the effective silencing of these genes that promote pathological change (Ballas et al., 2001). The effect of REST on cognition in AD has been shown using two different methods: Authors deleted REST in mouse models of AD, noting that it accelerated Aβ accumulation and the progressive buildup of misfolded and phosphorylated tau. Additionally, the authors used adeno-associated viruses (AAVs) to induce overexpression of REST in the hippocampus, suppressing Aβ and tau pathology (Aron et al., 2023). In healthy aging, cells induce REST expression to protect cells from age-related stress; however, in mild cognitive impairment and AD, REST manifests lower expression, likely contributing to more severe symptoms (Lu et al., 2014).

Additionally, overexpression of the gene ubiquitously transcribed tetratricopeptide repeat on chromosome X (KDM6A), which plays a role in gene expression as a demethylase and escapes X inactivation, improves Aβ-related cognitive deficits, cellular viability, and longevity (Tran et al., 2020). This effect has been mimicked with the addition of another X chromosome in mice, establishing a clear link between sex chromosomes and cognitive resilience to AD (Davis et al., 2020). Although no TF pathway has been linked to this effect, another genomic mechanism, namely, the modulation of chromatin accessibility caused by a lack of Xist RNA coating on the KDM6A region of the second X-chromosome, is likely what increases KDM6A expression (Davis et al., 2020).

#### 2.1.2 Resilience mechanisms to counteract hyperexcitability of neuronal networks

Increased neuronal hyperexcitability is prevalent during Alzheimer’s progression, leading to a disruption in neural circuits and contributing to synaptic loss, causing cognitive and memory decline. When prolonged, hyperexcitability can result in frank seizures and also directly induce neuronal cell death. In symptomatic AD, this manifests as an increased excitatory/inhibitory (E/I) ratio of electrical activity, with an overall net increase in cortical excitation, as seen in human electroencephalograms (EEGs) as well as in AD transgenic animal and cerebral organoid models (Lauterborn et al., 2021; Talantova et al., 2013; Palop and Mucke, 2016; Ghatak et al., 2019, 2021a,b; Li Z. et al., 2008; Ryan et al., 2013; Barker et al., 2021). While hyperexcitability is detrimental to neuronal networks, in contrast, homeostatic, physiological electrical activity-driven stimulation of transcription, in part due to Ca^2+^ signaling, regulates the level of gene transcription essential for memory, other neuronal functions, and resilience, e.g., as exemplified by the TFs CREB and MEF2C (Yap and Greenberg, 2018; Papadia et al., 2005).

MEF2C enhances cognitive performance and reduces cortical neuronal hyperexcitability by modulating genes involved in synaptic transmission and neuronal activity, including genes encoding sodium channels (Scn1a) and glutamate receptors (Gria4), increasing the expression of both (Barker et al., 2021). In the PS19 mouse model of tauopathy, researchers overexpressed MEF2C and observed a significant reduction in hyperexcitability, accompanied by improved cognitive flexibility (Barker et al., 2021). Moreover, MEF2C maintains stability in neuronal firing rates by adjusting synaptic connection strength to compensate for higher neuronal activity during AD (Barbosa et al., 2008). Not only does it play a role in signal strength, but it plays a role in enhancing signal reception and preventing neural degeneration, given that it binds to and upregulates immediate-early genes such as Egr1 and Arc, which promote dendritic spine density and complexity (Puang et al., 2020).

Interestingly, either too much or too little transcriptional activity due to mutation or changes in electrical activity (e.g., affecting MEF2C or MeCP2, a chromatin-associated protein that can either activate or repress transcription) can lead to dysfunction, particularly during development, with decreased MEF2C activity (MEF2C haploinsufficiency syndrome) or MeCP2 activity (Rett syndrome) resulting in severe cases of autism spectrum disorder/intellectual disability (Li H. et al., 2008; Lipton et al., 2009; Chahrour and Zoghbi, 2007). Notably, in contrast to the critical effect of MEF2C during development in facilitating neurogenesis and synapse formation, in mature neurons MEF2C activity leads to synaptic sculpting and elimination, important for synaptic plasticity (Flavell et al., 2006; Shalizi et al., 2006; Barbosa et al., 2008).

Moreover, the expression of RNA binding fox-1 homolog 1 (RBFOX1) has been reported to have an inverse relationship with cognitive decline in the human early AD brain; RBFOX1 modulates the alternative splicing of ion channels [including sodium voltage-gated channel alpha subunit 1 (SCN1A), calcium channel, voltage-dependent, L type, alpha 1C subunit (CACNA1C)], GABA receptor subunits, and genes involved in synaptic vesicle docking (Gehman et al., 2011; Vuong et al., 2018; Raghavan et al., 2020). Through a change in alternative splicing patterns and modulation of gene expression, RBFOX1 plays a critical role in suppressing the production of ion channels, receptor subunits and other targets linked to increased excitability. Note, however, that recent evidence suggests high-molecular weight tau inhibits bursting of neuronal action potentials in CA1 hippocampus, apparently by repressing expression of CaV2.3 calcium channels, also potentially disrupting memory function (Harris et al., 2025).

#### 2.1.3 Resilience mechanisms to counteract oxidative and nitrosative stress that contribute to synaptic damage in neurons

In the brains of AD patients, there are multiple reports of increased oxidative stress and nitrosative stress, which can cause a variety of chemical reactions to engage aberrant signaling pathways, increase DNA damage, and contribute to synaptic damage and neuronal cell loss (Robert and Wagner, 2020; Oh et al., 2024). In cognitively resilient individuals, this is countered by an upregulation in antioxidant capabilities.

For example, NRF2 is one of the key transcriptional regulators counteracting this increase. NRF2 works to protect against oxidative/nitrosative stress and inflammatory responses through the upregulation of multiple phase II enzymes, including the antioxidant enzymes heme oxygenase 1 (HMOX1), NAD(P)H quinone dehydrogenase 1 (NQO1), and the gene glutamate-cysteine ligase catalytic subunit (GCLC), which is involved in enzymatic synthesis of the antioxidant glutathione (GSH) (Reichard et al., 2007; Itoh et al., 1997; Gall Trošelj et al., 2022). Notably, decreased nuclear levels of NRF2 and perturbations in NRF2-regulated genes have been reported in AD/ADRD brains (Ramsey et al., 2007). Additionally, knocking out NRF2 in mouse models of AD exacerbated cognitive decline (Branca et al., 2017). Furthermore, NRF2 upregulates light chain 3 beta (LC3B) and sequestosome 1 (SQSTM1/p62), the former of which is a crucial component of autophagosomes, and the latter of which marks proteins for degradation by autophagy in multiple cell types (Jiang et al., 2015; Frias et al., 2020). In patients with symptomatic AD, nuclear translocation of NRF2 is impaired due to Aβ accumulation, which stabilizes NRF2’s interaction with Kelch-like ECH-associated protein 1 (KEAP1). This causes less dissociation of NRF2 from KEAP1, which leads to less NRF2 traveling into the nucleus, which is necessary for its transcriptional effects on the antioxidant response (Guo et al., 2021; Ramsey et al., 2007). This impairment reduces NRF2’s ability to increase antioxidant and anti-inflammatory gene activity, resulting in synaptic damage because of less protection of processes involved in synaptic plasticity and consequent memory formation (Brandes et al., 2021). While the antioxidant and anti-inflammatory activity of NRF2 may be important directly in neurons, it is currently thought that activation of this transcription factor pathway in regulating gene expression related to combatting oxidative/nitrosative stress plays a larger role in microglia and astrocytes, as discussed below.

Critically, avoidance of nitrosative/oxidative stress is critical for maintaining synaptic connections and thus avoiding cognitive decline in the AD brain (Oh et al., 2024). Another critical factor to maintain synapses and thus cognitive resilience from AD is the transcriptional regulator Zinc Finger CCHC-type containing 17 (ZCCHC17), which upregulates genes involved in synaptic function and whose expression is negatively correlated with tau tangle burden and positively correlated with resilience (Bartosch et al., 2024). Similarly, cAMP response element-binding protein (CREB), a regulator of expression of genes stabilizing synaptic changes during learning, plays a strong role in increasing synaptic plasticity. In mouse models of AD, reversing CREB deficiency decreases Aβ and p-tau231 and mitigates neuroinflammation, ultimately leading to increased synaptic plasticity and cognitive function (Rong et al., 2025). In mouse forebrain, increased CREB activity due to overexpression of Calcium/calmodulin-dependent protein kinase IV (CaMKIV) leads to higher contextual fear and social recognition memory accompanied by higher long-term plasticity in hippocampus and anterior cingulate cortex (Wu et al., 2008; Fukushima et al., 2008). CREB is also a positive regulator of long-term memory consolidation in the hippocampus, with patients that overexpress CREB exhibiting stronger and more stable memories (Kida et al., 2002; Kida, 2012).

More recent work has shown that actions of CRTC1, a coregulator of CREB activity, can be influenced by environmental factors, such as air pollution. Components of air pollution result in excessive nitrosative stress in the brain and consequent aberrant protein S-nitrosylation (-SNO) of many proteins, including a critical cysteine residue of CRTC1 (Oh et al., 2024; Zhang et al., 2025). This redox-mediated posttranslational modification inhibits CREB transcriptional activity and thus disrupts memory pathways in the hippocampus (Zhang et al., 2025). Mechanisms that combat nitrosative stress from air pollution, including the NRF2 pathway, abate these aberrant redox reactions and thus can contribute to cognitive resilience. Interestingly, activity of the resilience transcription factor MEF2C can also be inhibited by nitrosative or oxidative stress due to chemical modification (-SNO or SO_2/3_H, respectively) on a critical cysteine residue of MEF2C that affects its binding to DNA and thus inhibits its transcriptional activity (Ryan et al., 2013; Okamoto et al., 2014).

### 2.2 Microglia

Microglia are brain resident, innate immune system cells that function to remove extracellular debris, recognize pathogens and produce pro-inflammatory factors to signal invasion, and prune synapses in the central nervous system (Colonna and Butovsky, 2017). In cognitively resilient patients, microglia possess homeostatic functions that may mitigate neurodegeneration through clearance of misfolded proteins (i.e., Aβ plaques, tau tangles), in part via phagocytosis after transient activation of neuroinflammatory pathways (Das et al., 2020). In contrast, with the sustained inflammation that occurs in AD, microglia display what has been termed as a disease-associated microglia (DAM) phenotype (Lively and Schlichter, 2018; Rangaraju et al., 2018; Liu et al., 2025), and can contribute to pro-inflammatory damage in neurodegenerative disorders; for example, microglia begin to participate in complement-mediated phagocytosis and resultant synapse loss in the AD brain (Hong et al., 2016). Moreover, activation of complement factor 3 (C3) by aberrant S-nitrosylation in the human AD brain may trigger intense synapse loss in this manner (Yang et al., 2022).

Accordingly, ameliorating prolonged neuroinflammation can stave off, at least in part, synapse loss and neurodegeneration initiated by both aggregated Aβ and tau. One regulator that modulates neuroinflammation-related networks in this manner is retinoic acid-related orphan receptor alpha (RORα). RORα agonists are known to decrease reactive oxygen species, and reduce production of pro-inflammatory cytokines and signaling molecules such as interleukin 6 (IL-6) and nuclear factor kappa-light-chain-enhancer of activated B cells (NF-κB) (Oh et al., 2019; Mao et al., 2021).

Left unabated, however, microglial inflammation can result not only in synaptic loss but also lower homeostatic gene expression, and promotion of tau pathology spread (Wang et al., 2022; Wu C. et al., 2022; Keren-Shaul et al., 2017). Moreover, microglia begin to release pro-inflammatory cytokines, which cause disruptions in cell-cell communication and induce apoptosis in neurons (Fan et al., 2017). The microglial inflammatory response also has effects on the transcriptional networks of neurons. For example, in microglia, activation of the cyclic GMP-AMP synthase (cGAS)-Stimulator of Interferon Genes (STING) pathway and thus the pro-inflammatory cytokine interferon type 1 (IFN-I, often elicited by pathogenic tau) reduces MEF2C expression in neurons (Udeochu et al., 2023).

On the other hand, when MEF2C is highly expressed in microglia, it suppresses expression of IFN-I, which downregulates the microglial transition to the DAM state (Deczkowska et al., 2017). In cognitive resilience, this would drive the reduction of microglial synapse elimination (associated with the inflammatory state), preventing expansive loss of neuronal connections (Deczkowska et al., 2017). This increase in homeostatic microglia downregulates pro-inflammatory cytokines, increasing synaptic plasticity and reducing downstream neuronal cell death (Maher et al., 2005; Butovsky et al., 2005; Zhou et al., 2012). Recent data suggest that MEF2C may restrain microglial overactivation by inhibiting cyclin dependent kinase CDK2 (Hu et al., 2025). Microglial REST also reportedly represses the release of pro-inflammatory cytokines in microglia (Yu et al., 2020).

NRF2 activation in microglia during AD upregulates genes with an antioxidant effect, reducing oxidative stress burden, and exerting effects downstream of its elicited antioxidant response that reduce neuroinflammation (Innamorato et al., 2008). Note that NRF2 signaling is important for cognitive resilience not only in microglia, but also in astrocytes (see below), oligodendrocytes (see below), and possibly in neurons (as discussed above) (Satoh et al., 2008; Baxter et al., 2021).

### 2.3 Astrocytes

In the brain, astrocytes are glial cells and represent the most abundant non-neuronal cell type. Astrocytes function to maintain homeostasis in and around neurons, regulate the blood-brain barrier, provide metabolic support to neurons, and have recently been shown to manifest network signaling themselves (Sofroniew and Vinters, 2010; Cahill et al., 2024).

Reactive astrocytes are a subtype of astrocyte that become activated in response to diseased states of the brain, and can occupy one of two cell states, one being neurotoxic and the other being neuroprotective (Chun and Lee, 2018). Neurotoxic astrocytes secrete proapoptotic and proinflammatory signals that tend to further disease progression (Liddelow et al., 2017). This cellular state is often induced by microglial secretion of pro-inflammatory cytokines such as interleukin 1 alpha (IL-1α), tumor necrosis factor (TNF), and complement component 1q (C1q), as well as NF-κB signaling, providing another reason that lowered pro-inflammatory markers secreted by microglia might exert beneficial effects during resilience (Liddelow et al., 2017). Alternatively, astrocytes can also exert neuroprotective effects through protection of the blood-brain barrier, maintenance of synaptic transmission, release of neurotrophic factors, and resultantly, a reduction in neuronal death (Bush et al., 1999; Zamanian et al., 2012). This state is commonly induced by the activity of the TF, signal transducer and activator of transcription 3 (STAT3), a protein that mediates cellular growth and differentiation, and the development and maturation of the immune system (Hillmer et al., 2016; Anderson et al., 2016).

The balance between neurotoxic and neuroprotective astrocytes in part confers resilience. Higher levels of the latter increase synaptic strength and stability as well as aid in tissue recovery and repair (Gao et al., 2005; Zador et al., 2009; Hayakawa et al., 2014). Additionally, lowered numbers of neurotoxic astrocytes result in a decrease in neuronal cell death and synapse loss (Liddelow et al., 2017).

Moreover, the function of many astrocytes can be modified through several additional transcriptional networks. For example, NRF2 regulates the antioxidant response under oxidative or nitrosative stress, similar to its actions in other cell types (Shih et al., 2003). This has a large impact on improving mitochondrial function, given that reactive oxygen and nitrogen species can have a significant impact on mitochondrial structural stability and function (Nakamura et al., 2021). In symptomatic AD, nuclear translocation of NRF2 is inhibited, preventing its transcriptional function (Davies et al., 2021).

Furthermore, upregulation in REST reduces astrocyte reactivity through repression of inflammatory markers that segregate astrocytes into a reactive subpopulation, thus preventing neuroinflammation-induced reactive cells from populating the brain and exerting neurotoxic effects in human induced pluripotent stem cell (hiPSC)-derived astrocytes from patients with Down syndrome, a condition associated with early-onset AD (Huang et al., 2025).

### 2.4 Oligodendrocyte precursor cells and oligodendrocytes

Oligodendrocytes are glial cells that myelinate neurons in the CNS, aiding in rapid and efficient transmission of nerve impulses (Kuhn et al., 2019). Myelin is also critical for axonal protection and maintenance of cellular homeostasis. In cognitively normal individuals, lower myelin content is in fact associated with an increased rate of subsequent cognitive decline (Gong et al., 2023). Myelination is thought to contribute to prevention of cognitive decline through increases in neuronal energy efficiency and axonal protection from tau spread (Rubinski et al., 2022; Gerevich et al., 2023). Moreover, the spread of AD pathology throughout the brain is inversely proportional to the pattern of cortical myelination during development, suggesting that oligodendrocyte factors may delay neurofibrillary changes (Braak and Braak, 1996).

As expected, therefore, preserved myelination is associated with decreased cognitive decline in AD, correlating with decreased pathological misfolded protein buildup and increased cellular resistance to the effects of this buildup (Chen et al., 2021; Depp et al., 2023). There are several transcriptional networks involved in the maintenance of myelination that may contribute to this resilience. NRF2 has been shown to be important in oligodendrocytes and OPCs, with loss of this TF and resultant change in expression of affected genes apparently contributing to demyelination in some neurological conditions such as multiple sclerosis (Liessem-Schmitz et al., 2018; Nellessen et al., 2020; De Nuccio et al., 2020). Additionally, the network of genes affected by REST can contribute to myelination. For example, REST modulates chromatin accessibility at differentiation-related genes, recruiting histone deacetylases to repress the expression of genes preventing oligodendrocyte differentiation (DeWald et al., 2011). Furthermore, it represses the expression of neuronal genes in OPCs, increasing their capacity to focus on glial programs (DeWald et al., 2011). This has been shown experimentally as follows: When a dominant-negative form of REST was expressed in OPCs, they expressed more neuronal proteins, TFs, and marker antigens. Moreover, overexpression of REST in rat OPCs produced a higher rate of differentiation into pre-oligodendrocytes (DeWald et al., 2011). REST is demonstrably important in ensuring OPC differentiation, and as such may represent an important target in restoring myelination in AD brains.

## 3 Synthesis of transcriptional effects on specific cell types

### 3.1 Convergence of transcriptional regulation in cognitive resilience

Maintaining neurological function in the face of AD depends on continuous coordination between diverse cell types. Propelled by the TF-modulated production of proteins critical for their function, oligodendrocytes myelinate the axons of neurons; microglia clear debris and coordinate immunological responses to prevent disease and infection; astrocytes maintain homeostasis in and around synapses, provide metabolic support for neurons, and have their own signaling capabilities; and neurons communicate among themselves as well as with an expansive repertoire of glial cells (Kuhn et al., 2019; Colonna and Butovsky, 2017; Das et al., 2020; Sofroniew and Vinters, 2010; Cahill et al., 2024). Thus, a complex network of intercellular support keeps neurons and their synaptic contacts sufficiently stable so as to maintain function in the face of pathology. Other cell types such as pericytes, ependymal cells, and endothelial cells have not been included in the current analysis due to a lack of evidence implicating them in resilience, but they may well contribute.

Examining the transcriptional changes in all these cell types, there are a few convergent themes that emerge: Resilience depends on a collective effort to preserve synaptic integrity, suppress neuroinflammation, support myelination and axonal conductance, facilitate debris clearance, and maintain appropriate cellular repair standards and quality control (Bartosch et al., 2024; Rong et al., 2025; Das et al., 2020; Gong et al., 2023; Rubinski et al., 2022; Gerevich et al., 2023; Lively and Schlichter, 2018; Rangaraju et al., 2018; Davies et al., 2021).

**FIGURE 1:**
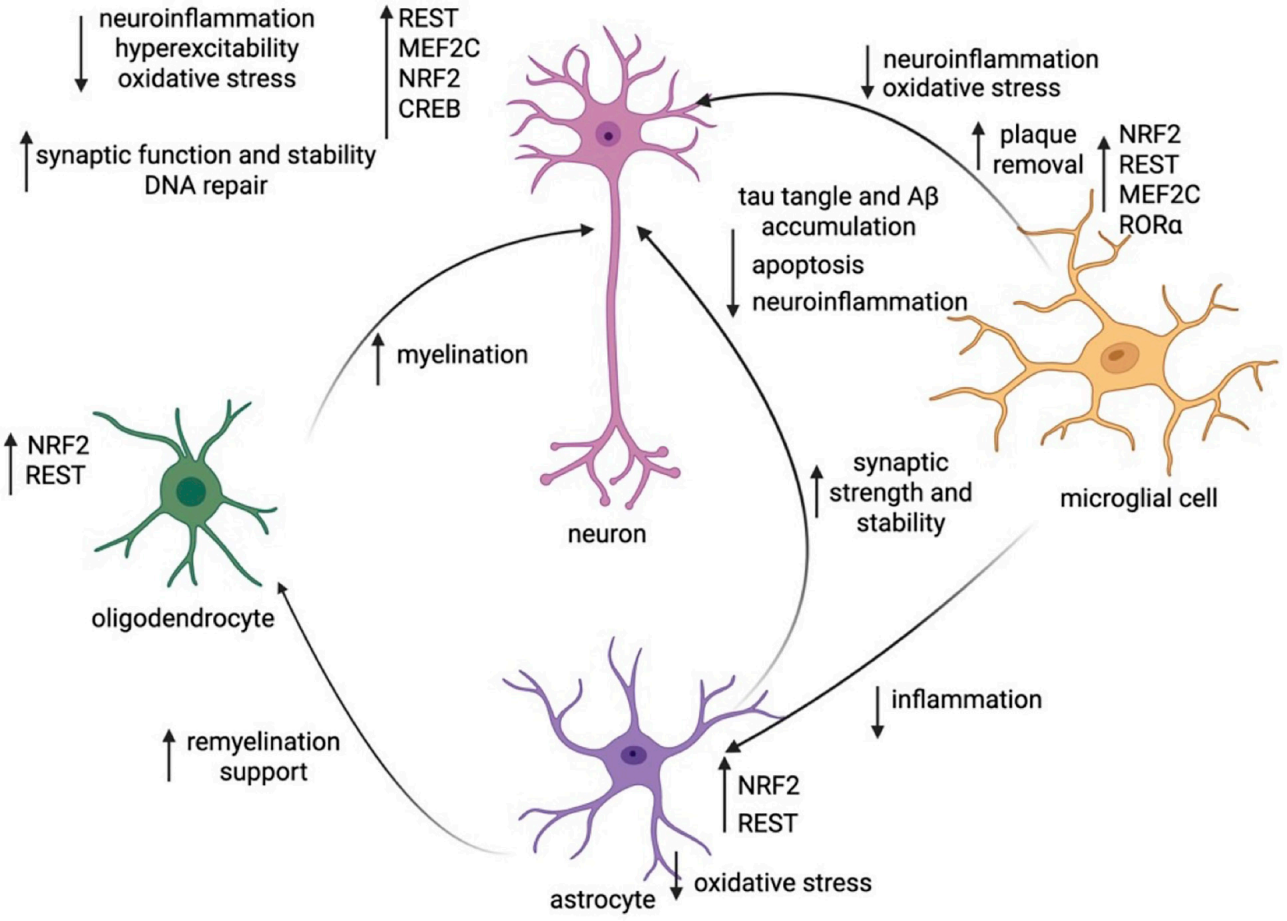
Working model of transcriptionally-regulated change within and between cell subtypes in cognitive resilience. Synthesis of the key TF changes within each cell subtype (neurons, microglia, astrocytes, oligodendrocytes) and the changes in cellular function they induce within all cell types. Created in https://BioRender.com.

In Figure 1, we present a working model of these cell-specific changes in transcription networks and how these shifts affect key neurological processes.

When impacts on different cell groups are considered on a TF-level, many TF actions are pleiotropic across cell types. MEF2C is critical for prevention of neuronal hyperexcitability through controlled suppression of neural activity, increased inhibitory neurotransmitter release, sculpting and refining synaptic endings, and strategic control of ion channel expression (Barker et al., 2021). MEF2C also prevents release of pro-inflammatory cytokines in microglia, reducing their likelihood of transition to a DAM state (Deczkowska et al., 2017). Interestingly, some TFs may demonstrate both cell autonomous and non-cell autonomous effects. For example, MEF2C reduces neuroinflammatory signaling in microglia, preventing microglia from switching to a DAM state; as a result, MEF2C plays an indirect role in preventing reactive astrocytes from neurotoxic actions, promoting OPC differentiation, and fostering remyelination by oligodendrocytes, all processes impacted by microglia-driven neuroinflammation (Butovsky and Weiner, 2018; Rangaraju et al., 2018; Liddelow et al., 2017; Starost et al., 2020; Zveik et al., 2024).

RE-1 silencing transcription factor also acts in multiple cell types. In neurons, it downregulates genes associated with pathogenic pathways (i.e., both those that are pro-apoptotic and those that are increasing Aβ and tau accumulation) (Aron et al., 2023). In microglia, it prevents release of pro-inflammatory cytokines, and similarly in astrocytes it represses the inflammatory markers that cause them to take on the neurotoxic phenotype (Yu et al., 2020; Huang et al., 2025). Moreover, in OPCs REST recruits histone deacetylases to repress genes that prevent differentiation, which increases myelination downstream (DeWald et al., 2011). Increased myelination by oligodendrocytes leads to higher neuronal energy efficiency and lower tau burden (Rubinski et al., 2022; Gerevich et al., 2023), and the repression of pro-inflammatory cytokines in microglia exerts downstream effects on reactive astrocytes that discourage transition to the neurotoxic state (Liddelow et al., 2017). Overall, due to a lowered number of disease-associated microglia and neurotoxic astrocytes, REST may play a role in reducing neurotoxicity (Huang et al., 2025; Edison, 2024).

NRF2 is another TF that acts directly in many cell types, though its functions are far more similar across different cells as opposed to REST, which exerts pleiotropic effects across cell types. Across neurons, astrocytes, and microglia, NRF2 acts to reduce oxidative/nitrosative stress, which has downstream effects on DNA damage and neuroinflammation (Ramsey et al., 2007; Innamorato et al., 2008; Robert and Wagner, 2020). The reduction in neuroinflammation will lead to downstream effects on astrocytes, decreasing their likelihood of conversion to a reactive state. In oligodendrocytes, NRF2 loss appears to reduce myelination in conditions such as multiple sclerosis, which could have impacts on neuronal synaptic communication and energy utilization (Liessem-Schmitz et al., 2018; Nellessen et al., 2020; De Nuccio et al., 2020; Rubinski et al., 2022; Gerevich et al., 2023).

MEF2C, REST, and NRF2 are TFs that play critical and distinct roles in conferring resilience to AD across many cell types. We therefore propose that they function as “master regulators” of cognitive resilience to AD and warrant more investigation. Interestingly, there is also crosstalk between master regulators, e.g., whereby increased MEF2 isoforms can upregulate NRF2, implying that resilience networks may mutually drive each other (Nagar et al., 2017). In human patients with AD, knockdown of MEF2C downregulates expression levels of the components of the NRF2-ARE pathway (Ren et al., 2022), implying that resilience networks may collaborate to synergistically alter disease progression.

Several TFs also play more minor cell autonomous roles with some non-cell autonomous features. First, ZCCHC17 preserves synaptic homeostasis in neurons. Its expression is negatively correlated with tau tangle burden and positively correlated with resilience; however, it does not exert any discernible non-cell autonomous effects (Bartosch et al., 2024). CREB also plays a major role in synaptic plasticity in neurons, as a regulator of neuronal genes stabilizing synaptic changes during learning. In a mouse model of AD, reversal of CREB deficiency decreased Aβ and p-tau231, and thus reduced neuroinflammation (Rong et al., 2025). Mitigation of neuroinflammation likely has impacts on reducing microglial transition to a DAM state and possibly decreases astrocyte neurotoxicity and increases OPC differentiation (Starost et al., 2020; Chew et al., 2005; Liddelow et al., 2017; Rangaraju et al., 2018). RORα acts directly upon microglia to reduce neuroinflammation, potentially adding to the anti-inflammatory effect of MEF2C and CREB (Oh et al., 2019; Mao et al., 2021).

### 3.2 How are transcriptional regulators engaged to facilitate resilience?

Transcription factors are in part engaged to promote cognitive resilience to AD through gene by environment (GxE) interactions that are dependent on (1) genetic variants and (2) lifestyle and environmental factors. Multiple single-nucleotide polymorphisms (SNPs) associated with resilience have been described (O’Neill et al., 2024; Dumitrescu et al., 2020). It is known that, generally, genetic SNPs play a role in eliciting transcriptional change (Van Dongen et al., 2016). For instance, variants in regulatory regions can directly affect the ability of certain TFs to bind (Johnston et al., 2019). Additionally, a large number of genetic variants in humans might impact methylation of specific cytosine residues that confer epigenetic repression of gene transcription (Van Dongen et al., 2016). Furthermore, increases in copies of a gene tend to increase dosage of product produced (Pollack et al., 1999). Nonetheless, there is no substantive evidence showing that SNPs associated with resilience directly cause transcriptional changes, so SNP-transcription associations will not be further reviewed here.

Lifestyle factors and the environment may also be implicated in control of transcription. The environment is known to be impactful in modulating the expression of transcription networks to confer certain traits (Sturtevant, 1913). There is also potential reason to believe that GxE interactions impact downstream resilience to AD. One study reviewed these interactions in mice, and found a locus on chromosome 10 that appears to increase cognitive resilience to AD in female mice, and whose impact is strengthened when mice are fed a high-fat and high-sugar diet (Dunn et al., 2025). Overall, there is growing evidence suggesting the expression level of TFs implicated in resilience is associated with lifestyle changes.

For RBFOX1, a key splicing factor implicated in synaptic function that may play a role in cognitive resilience to AD, lifestyle exposures such as physical activity and diet have been linked to changes in expression (Gehman et al., 2011; Vuong et al., 2018; Ahn et al., 2024; Black et al., 2017; Raghavan et al., 2020). Acute physical stress leads to diversification of the transcriptome in mouse skeletal muscle in RNA-binding proteins such as RBFOX1 (Ahn et al., 2024). Additionally, a high-fat diet was shown to shift alternative splicing in skeletal muscle, potentially implying a role for RBFOX1 (Black et al., 2017).

MEF2C, a TF found in the brain exclusively in neurons and microglia, was first discovered by one of the authors (S.A.L., see Leifer et al., 1993). This TF is critical to neural development and function, but also moderating neuronal hyperexcitability and synaptic function and stability in AD and possibly in normal aging (Barbosa et al., 2008; Barker et al., 2021). Enriched environments, which include increased physical, social, and cognitive activity, were shown to increase MEF2C expression and hence of its target genes (Barker et al., 2021). Additionally, poor air quality significantly reduces the expression of MEF2C (Akbar Esteki-Uoregan et al., 2019), as well as potentially mediates its inhibition by aberrant protein S-nitrosylation at Cys39, thus interfering with its DNA binding function (Ryan et al., 2013; Okamoto et al., 2014). Moreover, sleep loss in mice leads to an increase in MEF2C dephosphorylation in the cortex, thus contributing to increased transcriptional activity (Bjorness et al., 2020).

RE-1 silencing transcription factor, a TF that represses neural genes in non-neuronal cell types, downregulates genes involved in pathogenic pathways, reduces inflammation, and increases remyelination in the brain (Aron et al., 2023; Huang et al., 2025; DeWald et al., 2011). In mice, both consuming a Western diet and increasing physical activity have been shown to increase expression of REST (Medford et al., 2014; Dallagnol et al., 2017).

cAMP response element-binding protein is a TF that promotes learning and memory through enhancement of synaptic stability during learning. In adult male mice, 6 weeks of exercise intervention significantly activated the cAMP/PKA/CREB pathway (Jin et al., 2024). Also in mice, high levels of particulate matter in the air have been reported to decrease CREB expression (Li et al., 2024). Learning has also been shown to lead to an increase in CREB expression in mollusks (Sadamoto, 2010). CREB phosphorylation is also increased over the course of REM sleep, leading to increased CREB activity (Luo et al., 2013). In adult rodents, social isolation decreases CREB expression, though this can be reversed by antidepressant treatment (Wallace et al., 2009).

NRF2 is a TF that binds the antioxidant response element and upregulates antioxidant and anti-inflammatory genes (Branca et al., 2017). In response to two consecutive days of acute exercise stress, wild-type mice experienced upregulation of NRF2 (Muthusamy et al., 2012). Additionally, the bioactive compound curcumin found in various food items increases the amount of NRF2 protein and expression of downstream genes in mouse cortical neuronal cells, enhancing antioxidant defense (Park et al., 2021). However, drugs such as curcumin are quinones, known to be active electrophiles that in the high doses needed for NRF2 activation can also manifest serious off-target, systemic side effects (Satoh et al., 2013). In contrast, in multiple mouse AD/ADRD models, an innocuous pro-drug, which is only converted to an active quinone locally by the oxidation and inflammation that it then combats, has been shown to be a robust NRF2 activator. The clinically-tolerated pro-drug, carnosic acid or its improved di-acetylated derivative, has been shown to be beneficial not only at the level of reducing Aβ and tau pathology, but also in improving inflammation, synaptic number, and neurobehavioral tests (Lipton et al., 2016; Banerjee et al., 2025).

Interestingly, increased particulate matter upregulates the NRF2-ARE pathway, likely to counteract the increase in oxidative stress (Li et al., 2000). There is also a noted link between NRF2 and sleep, where circadian cycle disruption in mice results in a downregulation in NRF2 (Lee et al., 2013). Finally, enriched environments (e.g., socially, physically, and cognitively enriched) have been reported to upregulate the NRF2-ARE pathway in rats (Zhang et al., 2021).

The TF RORα prevents pro-inflammatory signaling and oxidative stress, as well as regulates the circadian rhythm. In rodents, its expression is upregulated by an increase in VO_2_ max (Smith et al., 2023). Additionally, it is induced in response to the increased inflammation created by adipose tissue, promoting the presence of pro-inflammatory macrophages (Hams et al., 2020). Sleep restriction also causes downregulation in RORα in human blood cells, which further cements its association with the sleep-wake cycle (Möller-Levet et al., 2013).

**FIGURE 2:**
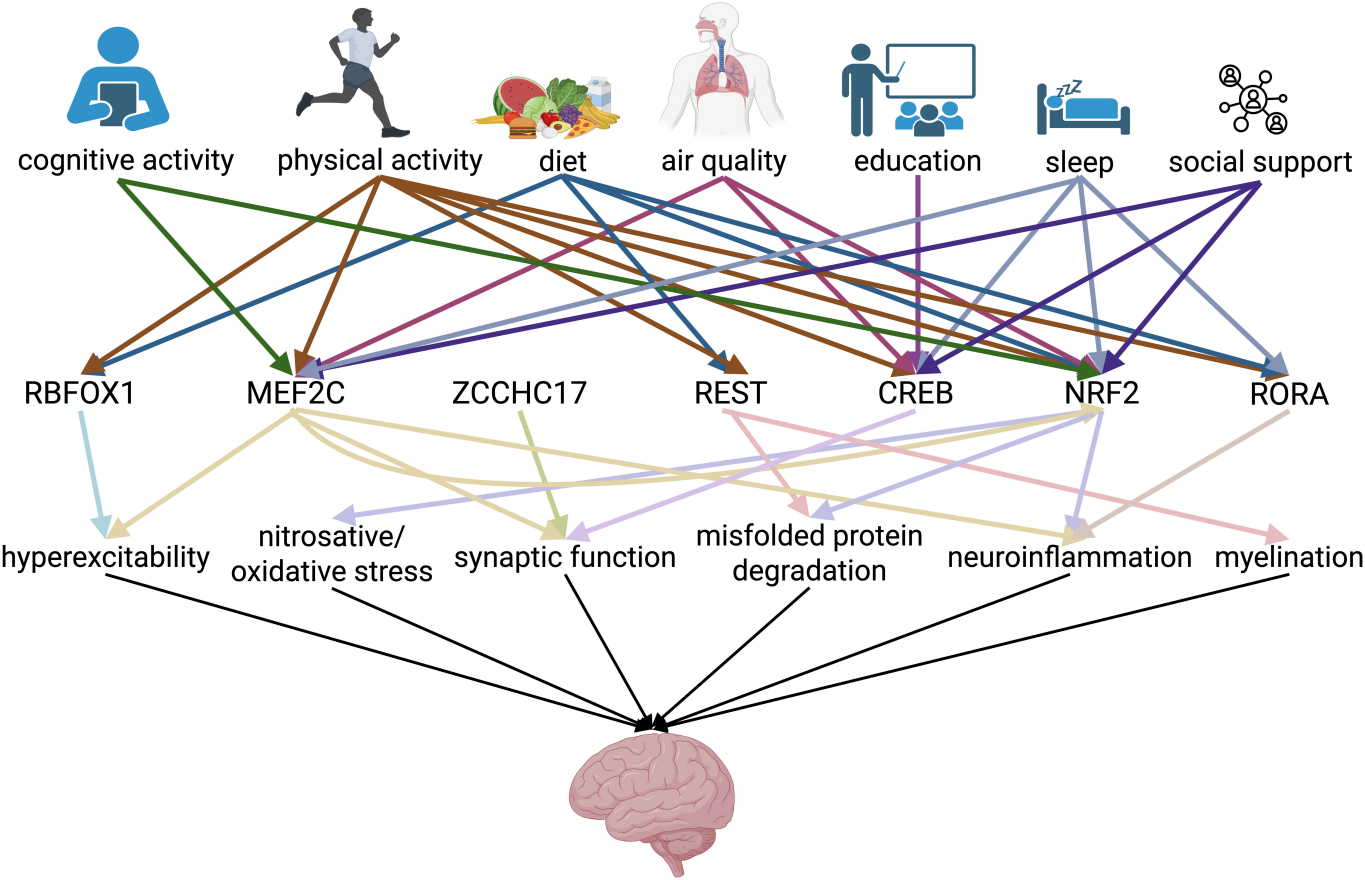
Working hierarchical model of risk factor-transcriptional regulator-neurological function interaction in conferring cognitive resilience. Schema showing the interaction among several layers of factors conferring cognitive resilience, encompassing lifestyle changes and transcription factors, leading to preserved neurological function. Arrows represent a link identified between the pair of factors as supported in the literature. Color of arrows in first row correspond to lifestyle factor, and color of arrows in second row correspond to transcription factor. Created in https://BioRender.com.

Given these identified links, we can create a hierarchical model for how upstream environmental and activity-related influences impact transcriptional networks which ultimately influence several drivers of cognitive function (Figure 2).

Here, we identify mechanistic links between most of the major lifestyle predictors of cognitive resilience and specific transcription factors that are implicated in cognitive resilience. This integrative model helps explain why factors previously treated as independent correlates such as education, physical activity, and diet consistently predict resilience; they likely converge on transcriptional regulators such as MEF2C, NRF2, CREB, REST, and RORα that counter the impact of pathological changes. More work on the elucidation of how environmental and social factors recruit biological mechanisms to afford neuroprotection will undoubtedly shed novel light on additional regulatory mechanisms that enable resilience.

## 4 Areas of future research

This review highlights the pivotal role of transcriptional regulators across a number of cell types in maintaining the integrity of organismal health in individuals who remain cognitively healthy despite the presence of some feature of AD pathology. Our synthesis of current data indicates that resilience benefits from the convergence of anti-inflammatory, pro-synaptic, debris-clearing, anti-apoptotic, and antioxidant transcriptional programs across various cell types. Evidently, resilience is not simply achieved through the mere prevention of pathology, but through true transcriptional adaptation that preserves function in the presence of pathology.

Our working model of transcriptional change in cognitive resilience (illustrated in Figure 1) shows how transcriptional regulators act in unison across various cell types to mediate resilience. There are several areas of future research that may further elucidate the mechanisms driving resilience in different human populations, including sex differences in and temporal evolution of transcriptional regulation.

### 4.1 Sex differences in the transcriptional regulation of cognitive resilience

The role of biological sex in mediating cognitive resilience to AD remains an underexplored area. Notably, nearly two-thirds of AD patients are women; however, men manifest higher prevalence and earlier onset of mild cognitive impairment (MCI) than women, at least in some populations (Rajan et al., 2021; Jack et al., 2019; Petersen et al., 2010; Roberts et al., 2012). Several genes on sex chromosomes are linked to resilience, with the X chromosome-situated histone deacetylase KDM6A being the most notable example. KDM6A is a gene that escapes X inactivation in mouse and humans, and whose overexpression improves Aβ-related mortality, cognitive deficits, and cellular viability (Davis et al., 2020; Neuner et al., 2022). As a regulator of gene expression, its protective effects are likely due to its role in modulating the transcription of resilience-associated genes. AD mouse models with an XX genotype (i.e., females) have greater cognitive reserve as a result; moreover, higher KDM6A expression is associated with cognitive resilience in female humans as well (Davis et al., 2020).

Intriguingly, the TFs that modulate resilience often show sex-specific effects in non-diseased models. For example, MEF2C seems to show greater impact on bone metabolism and altered social behaviors and prefrontal cortex inhibitory transmission in females (Maisuria et al., 2023; Cho et al., 2024). There is currently little research available on sex differences in TF action regarding cognitive resilience, and as such we do not fully understand whether those changes in TF efficacy are relevant to cognitive resilience.

However, many studies have shown significant sex-related differences in differentially-expressed genes (DEGs) and their enrichment analyses. In one study examining the superior frontal gyrus of symptomatic AD vs. resilient patients, genes linked to translation, autophagy, and heat shock proteins were more strongly upregulated in resilience in males compared to resilience in females. However, in that same study, female resilient patients manifested stronger upregulation of genes related to interferon signaling, mitochondrial processes, and metallothionein compared to males (de Vries et al., 2024b). Moreover, although not specific to AD, another report quantifying gene expression changes in the hippocampus, entorhinal cortex, superior-frontal gyrus, and postcentral gyrus over the course of brain aging showed strong sexually-dimorphic changes. They found that genes aiding energy production and protein synthesis/transport were significantly downregulated in the male aging brain, whereas females displayed stronger increases in immune activation with age compared to males (Berchtold et al., 2008).

This area of sex-specific changes merits future research to facilitate personalized interventions to slow AD-related cognitive decline. Specifically, we recommend conducting studies on the sex-specific impact of TFs relevant to cognitive resilience on protection from AD, as well as gene expression analyses to identify other candidate TFs that may play a sex-limited role. In a similar vein, examining the relationship among other population variables (ethnicity, co-morbidities, etc.,) and cognitive resilience might also provide another lens through which we can better understand resilience and related targets.

### 4.2 Temporal dynamics in transcriptional regulation in cognitive resilience

Mounting evidence suggests that transcriptional profiles vary with each stage of AD, with most networks shifting toward a neuroprotective state early on, and later transitioning to an increasingly neurotoxic one. For example, microglia follow this pattern, where increasing numbers transform from homeostatic to DAM as AD progresses (Keren-Shaul et al., 2017). Moreover, deactivation of the DAM transcriptional profile in microglia by inhibiting HDAC6 in female mice led to attenuated synapse loss, increased mitochondrial homeostasis, and, most importantly, increased cognitive function (McAlpin et al., 2022). Thus, reducing DAM plays a role in attenuating cognitive damage. Part of this may be attributable to the effects of microglia alone, but another part is likely due to their downstream effects on other cell types; DAM release pro-inflammatory cytokines, leading to a lowered number of reactive neurotoxic astrocytes and increased oligodendrocyte myelination capacity (Lively and Schlichter, 2018; Rangaraju et al., 2018; Liddelow et al., 2017, Starost et al., 2020; Rubinski et al., 2022; Gerevich et al., 2023).

Additionally, increased synaptic pruning and loss during aging leads to decreased long-term potentiation, an electrophysiological correlate of learning and memory (Lu et al., 2024). In the AD brain, with these losses in synaptic integrity as well as changes in protein expression and excitatory/inhibitory imbalance, neural networks devolve toward a state of hyperexcitability (Lauterborn et al., 2021; Ghatak et al., 2024). In the presence of AD pathology with increasingly dysfunctional systems over the course of the disease, the action of cognitive resilience transcription factors like MEF2C, RBFOX1, ZCCHC17, and CREB may help neurons maintain healthy and plastic synaptic connections and avoid hyperexcitability (Barker et al., 2021; Vuong et al., 2018; Bartosch et al., 2024; Kida, 2012).

One major drawback in our understanding of the temporal progression of resilience is that there are not enough data directly assessing the temporal trajectories and evolution of single-cell transcript expression and cell type behavior during cognitive resilience to AD. While there is evidence surrounding the temporal landscape of microglial activation and neuronal hyperexcitability in resilience, conclusions related to astrocytes and oligodendrocytes are speculative based on known cell-cell interactions and should be further validated. Therefore, we suggest the need for longitudinal studies on both cell-type behavior (e.g., numbers reflecting DAM-signature microglia, neurotoxic vs. neuroprotective astrocytes, rate of OPC differentiation, neuronal hyperexcitability) and related transcriptional regulator activity during cognitive resilience versus symptomatic AD. Such data would significantly advance our understanding of how resilience emerges and is maintained.

### 4.3 Links to broader cognitive aging themes

All individuals who cognitively age, even if they have no overt disease, will experience some form of cognitive decline. However, there is strong variability in the severity and course of this decline in the “healthy aging” population. Many older adults develop mild cognitive impairment (MCI), which is defined as a greater level of deterioration of memory, attention, and cognitive function than expected for age. MCI converts to dementia at a rate of 10%–15% per year (Bruscoli and Lovestone, 2004). However, on the other end of the spectrum, many individuals remain mostly cognitively intact, experiencing minimal decline (Manly et al., 2022).

Strikingly, resilient individuals may exhibit abundant amyloid plaques at postmortem examination but little cognitive decline and in some cases less spread of tau pathology (Mormino and Papp, 2018; Llibre-Guerra et al., 2025). In an effort to begin to explain this phenomenon, normal cognitive aging may be linked in part to gene expression changes occurring in AD. For example, one study found that 93% of the genes that are differentially expressed in *Drosophila* with tau pathology vs. controls are also differentially expressed during cognitive aging (Wu T. et al., 2023). In particular, the activity of master regulators of cognitive resilience identified earlier (i.e., MEF2C, REST, and NRF2) may contribute to this variability. These TFs are also found to be reduced in expression with normal cognitive aging but if upregulated are associated with higher cognitive maintenance through reduced hyperexcitability, increased synaptic plasticity, and lowered nitrosative/oxidative stress (Wu J. et al., 2023; Zullo et al., 2019; Mao et al., 2019; Zhou et al., 2018).

Hence, better understanding the overlap between transcriptional changes in cognitive resilience to AD and cognitive resilience to normal aging might help us target cognitive decline more broadly in aging populations. In this context, cognitive resilience to aging might imply a level of cognition atypically high for a patient’s advanced age. Researchers can compare gene expression changes among brain tissue samples from cognitively-aged (non-diseased) adults who have cognition atypical for their age, cognitively-aged patients with expected cognition, patients resilient to AD, and patients positive and symptomatic for AD, analyzing the differences to better understand what processes are enriched in both “resilience” situations (AD vs. aging) and what transcription factors might be driving this. This approach would help elucidate whether treatments preserving cognitive function in the face of AD pathology may also be leveraged to preserve cognition in the broader non-diseased aging population.

### 4.4 A novel framework for translation

Despite the growing appreciation of transcriptional regulators that preserve function in the face of significant AD pathology, the mediators of cognitive resilience have not been leveraged clinically as biomarkers or therapeutic strategies for AD, ADRD, or general cognitive health.

There is likely to be substantial clinical utility in the development of biomarkers for cognitive resilience in order to stratify patient populations and/or track progress in clinical trials targeting AD, as well as for AD diagnostic tools. While this has not previously been accomplished, future research to establish a set of reference biomarkers to better identify individuals that harbor pathological changes linked to AD but are clinically asymptomatic would be of great value to the field. For example, measuring biomarkers of synapse number (e.g., SV2A PET) as well as the transcriptional activity of the molecular regulators of synaptic maintenance might yield more information regarding clinical course and trajectory over current measurements of pathological burden.

From a therapeutic perspective, as an alternative approach to directly addressing abnormal protein pathology, we suggest considering methods that stave off cognitive decline by altering resilience-promoting transcriptional networks. For example, clinically-tolerated small molecule (drug) treatments for NRF2 activation are discussed above. Additional methods to increase activity of resilience-promoting TFs, such as adeno-associated viruses (AAVs) or the Clustered Regularly Interspaced Short Palindromic Repeats (CRISPR)-Cas9 platform might afford novel avenues of treating neurodegeneration. For example, REST has previously been overexpressed with AAVs, and shown to suppress Aβ and tau pathology in AD mouse models (Aron et al., 2023). Along these lines, overexpression of NRF2 using AAV improves recovery and protection from toxicity in animal models of oxidative stress, implying that there is a strong clinical basis for the benefits of resilience-related TF overexpression (Liang et al., 2017).

Additionally, determining and addressing environmental drivers of resilience would aid in more indirect but more tractable targeting of these master regulators and their downstream targets. Research should be performed to update and validate the hierarchical model (Figure 2) presented in this review. Along these lines, we suggest replicating the approach of the FINGER study (Kivipelto et al., 2013) while measuring markers of resilience along with transcriptional and proteomic/metabolomic data to determine whether these lifestyle interventions make a strong difference in cognitive outcomes. These types of findings, if robust, would lead to changes in health and safety regulations. Collectively, in the future targeting these transcriptional regulators could provide a novel way to target cognitive decline and treat the devastating symptoms of AD/ADRD.

### 4.5 The role of the periphery in mediating resilience

There is a growing understanding that organ systems outside the CNS, that is, in the periphery, demonstrate significant dysfunction in the context of AD/ADRD (Cao and Zheng, 2018; Brenowitz et al., 2022; Sohrabi et al., 2022). In this regard, emerging data in animal studies highlight that AD pathology results in gene expression changes in the peripheral immune system and organism-wide (Ramakrishnan et al., 2024; Park et al., 2025).

Until very recently, however, no studies had investigated differential trajectories of these peripheral derangements in the context of cognitive resilience, or whether peripheral processes and systems might be leveraged to promote cognitive resilience. Recently, a potential association between clonal hematopoiesis of indeterminate potential (CHIP), a premalignant expansion of hematopoietic stem cells, and protection from AD was described in a large population-based cohort. CHIP is associated with non-germ line mutations in stem cell lineages. In this study CHIP-associated mutations in hematopoietic stem cells of several genes, including those encoding the enzymes DNA methyltransferase 3 alpha (DNMT3A) and tet methylcytosine dioxygenase 2 (TET2), were found to be associated with protection from AD (Bouzid et al., 2023). These enzymes play important roles in DNA methylation/demethylation and epigenetic regulation, especially in hematopoietic stem cells and immune cells to regulate adaptive and innate immunity in the periphery, possibly also affecting immune cells migrating into the CNS. As discussed above, such mutations exert epigenetic effects on gene transcription (Davletgildeeva and Kuznetsov, 2024; Yang et al., 2024). Interestingly, recent work in AD mouse models demonstrated that transplantation of TET2-mutant, but not DNMT3A-mutant, bone marrow-derived cells reduced cognitive decline and Aβ production, suggesting a causal association between TET2-mutations, clonal hematopoiesis, and cognitive resilience (Matatall et al., 2025).

While there has been some work linking systemic genetic mutations to resilience, future work should further investigate how gene expression programs and transcriptional regulation in peripheral systems known to engage in CNS-crosstalk (i.e., the hematopoietic niche, immune system, and gastrointestinal tract) might facilitate neuroprotection and promote cognitive resilience. If biomarkers for resilience are substantiated, as mentioned earlier, studies on gene expression and transcriptional enrichment in the periphery in patients positive for these biomarkers might add useful mechanistic insight to the study of cognitive resilience to AD. Additionally, for longitudinal studies in which resilient patients are identified, we propose studying multiple sets of non-neurological gene expression data and related proxies.

## 5 Concluding remarks

The concept of cognitive resilience highlights the wide gap between the presence of AD Aβ pathology and the clinical signs and symptoms of AD, which can be explained at least in part by adaptation across different cell types facilitated by changes in transcriptional regulation. As explored in this review, transcriptional changes within neurons in resilient patients prevent hyperexcitability, maintaining synaptic integrity, and reducing Aβ and tau tangle buildup. Multiple types of glial cells also contribute complementary roles: Microglia maintain immune homeostasis, astrocytes support metabolic and redox balance, and oligodendrocytes preserve myelin integrity through transcriptional adaptations. It seems that across all cell types, the master regulators REST, MEF2C, and NRF2 enhance pathways conferring overall cognitive function across cell types, including Aβ and tau tangle degradation, anti-apoptosis, antioxidant, and anti-inflammatory pathways contributing to synaptic health.

Pinpointing changes in gene expression help give insight into the upstream regulators that orchestrate multi-system responses conferring resilience. Transcription factors are dynamic, adaptable, and convenient convergent points of intervention, making them promising therapeutic targets. By shifting focus from prevention of pathology to the creation of cognitive resilience, transcriptional biology provides a transformative path toward preserving cognition in the face of AD and the broader array of aging-associated neurodegenerative disorders. With further exploration, cognitive resilience may no longer be considered the edge case, but the blueprint for future interventions to prevent AD.
